# Supplementary material for: Enabling low-cost and robust essentiality studies with high-throughput transposon mutagenesis (HTTM)
Source: PLoS One. 2023 Apr 11;18(4):e0283990. doi: 10.1371/journal.pone.0283990 (PMC10089323; doi:10.1371/journal.pone.0283990)
Supplement: S1 File — (PDF) [file pone.0283990.s001.pdf]

CIG7UBZN

WORKS FOR ME 1

HTTM : Mutagenesis V.(cig7ubzn)

Antoine Champie[1], Amélie De Grandmaison[1]

[1]Université de Sherbrooke

Antoine Champie

COMMENTS 0

ABSTRACT

Part one of the HTTM protocol. A low-cost and high-throughput Tn-seq protocol.
This part cover the transposon mutagenesis and selection steps.

PROTOCOL INFO

Antoine Champie, Amélie De Grandmaison . HTTM : Mutagenesis . **protocols.io**
https://protocols.io/view/httm-mutagenesis-cig7ubzn

KEYWORDS

HDTM, TnSeq, HTTM

CREATED

Oct 27, 2022

LAST MODIFIED

Nov 05, 2022

PROTOCOL INTEGER ID

71935

MATERIALS TEXT

Main materials used in this protocol. Exhaustive list available in the twinned publication.

A	B	C
Aluminum cover	Ultident	PS-FOILP-100
Deep well	Greinerbio-one/Fisher	780271-FD
p200 tips	Sarstedt	70,303
p300 tips	Sarstedt	70,304
200µl 96 well plate	Ultident	87-C96-NS
Dilution plate	fisher	12-556-008
Petri dish	SARSTEDT	82,1473,001
LB Broth	Biobasic	SD7002

Antibiotics and additives concentrations used in this protocol :

A	B
Ampicilin	100ng/µl
Spectinomycin	100ng/µl
2,6-Diaminopimelic acid	55ng/µl

BEFORE STARTING

Per plate refers to the number of 96 well plates of target cells that need to be processed.

Day 1

3m

1 (1-A) Make a 15 mL LB (Diaminopimelic acid [Dap], Ampicillin [Amp], Spectinomycin [Spec]) pre-culture (2 mL per plate minimum) of the donor strain eAC494 and incubate with agitation at 37 °C overnight.

2 (1-B) Prepare the 96 deep-well plates for conjugation :

2.1

10m

- Preheat the deep-well plates at 60 °C in a sterile incubator for 00:10:00
- Prepare 50 mL of LB-Agar for each plate and keep it above 70 °C

2.2 Using a multichannel pipette transfer 300 µL of molten LB-Agar in each well of the deep-well plates, paying attention not to create bubbles by keeping the tips on the side of the wells and not dispensing all the liquid.

2.3 Let dry in a biological hood for 3 days or until well dried but not cracked. (Optional : can be placed on a

heating mat set at 30 °C to shorten the drying time to 2 days).

Day 2

3 (2-A) Prepare a 500 mL LB (Dap, Amp, Spec) culture of the donor strain per plate by making a 1/250 dilution of the pre-culture and incubate overnight at 37 °C .

4 (2-B) Fill the deep-well plates with chosen medium (1.5 mL per well) and inoculate each well with the recipient strains. Incubate overnight at 37 °C .

20m

Day 3

10m

5 (3-A) Pellet the donor strain by centrifugation 6000 x g, 00:10:00 and discard the liquid.

6 (3-B) Resuspend the pellet in 10 mL LB per plate.

7 (3-C) Dispense 100 µL of concentrated donor culture into each recipient well.

10m

8 (3-D) Pellet cells by centrifugation 4000 x g, 00:10:00 and remove the supernatant with the Aspir-8 + 50 µL guide.

8.1 If not using the Aspir-8 + 50 µL guide, remove all supernatant and add 50 µL of LB to each well.

9 (3-E) Resuspend by agitating on a shaker 900 rpm, 00:10:00 and do a quick spin to recover all the cells at the bottom of the plate.

1h

10 (3-F) Take 50 µL from the resuspended cells and deposit them on the dried agar at the bottom of the

prepared deep-well plate. Let dry 01:00:00 in a biological hood and cover with a gas permeable plate seal.

2h

11 (3-G) Incubate the deep-well plates 02:00:00 at 37 °C for conjugation.

12 (3-H) Add 400 µL of selection media to each well and resuspend by agitating on a shaker at 900 rpm, 00:10:00 and do a quick spin to recover all the cells at the bottom of the plate.

13 (3-I) Transfer 250 µL of the resuspended cells to a new deep-well filled with 1500 µL of selection media (with antibiotics to select for newly obtained mutants). Cover with a gas permeable plate seal and incubate with agitation at 37 °C overnight.

14 (3-J)/(3-K) (Optional) Using 20 µL of the conjugation mix make serial dilutions and spot on selective plates to estimate the number of mutants obtained per well.
Selection markers :
- Donor strain : Dap, Amp, Spec
- Recipient : Target-dependant
- Transposon mutants : Target-dependant + Spec

Days 4 to 7

15 Make a passage from the previous plate to a new deep-well plate filled with selective medium.
The volume of the passage (optimized to pass 3 million mutants in *E.coli*) varies from day to day :

- 200 µL of day 4 (4-A)
- 100 µL on day 5 (5-A), 6 (6-A) and 7 (7-A)

16 (7-B) (Optional) In order to have a backup in case of an issue during DNA extraction, make a glycerol stock using 150 µL of the culture after the passage, and store it at -80 °C .

10m

Day 8

10m

17 (8-A)/(8-B) Pellet cells by centrifugation 4000 x g, 00:10:00 and remove the supernatant. Aspir-8 can be used to accelerate this step. Cells are ready for DNA extraction and can be stored at -80 °C until ready to process.

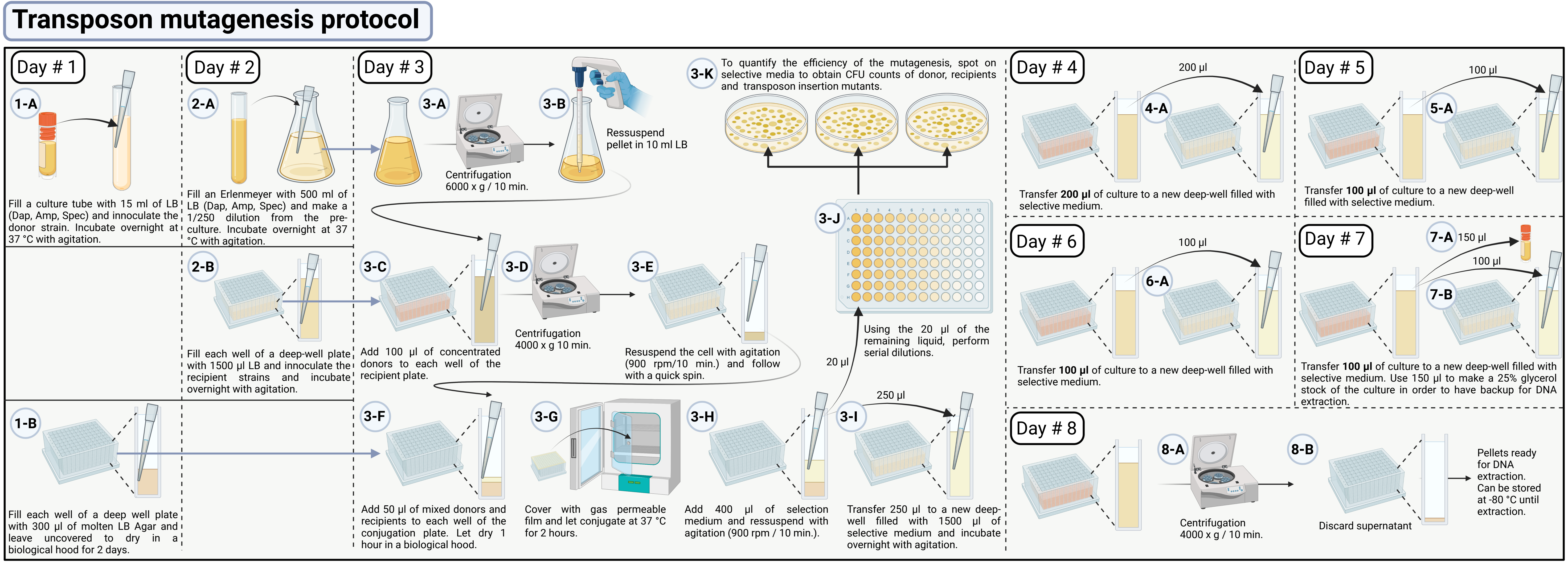

Transposon mutagenesis protocol
Donor
Recipients
Conjugation plate
Day # 1
1-A
Fill a culture tube with 15 ml of LB (Dap, Amp, Spec) and innoculate the donor strain. Incubate overnight at 37 °C with agitation.
1-B
Fill each well of a deep well plate with 300 µl of molten LB Agar and leave uncovered to dry in a biological hood for 2 days.
Day # 2
2-A
Fill an Erlenmeyer with 500 ml of LB (Dap, Amp, Spec) and make a 1/250 dilution from the pre-culture. Incubate overnight at 37 °C with agitation.
2-B
Fill each well of a deep-well plate with 1500 µl LB and innoculate the recipient strains and incubate overnight with agitation.
Day # 3
3-A
Centrifugation
6000 x g / 10 min.
3-B
Ressuspend pellet in 10 ml LB
3-C
Add 100 µl of concentrated donors to each well of the recipient plate.
3-D
Centrifugation
4000 x g 10 min.
3-E
Resuspend the cell with agitation (900 rpm/10 min.) and follow with a quick spin.
3-F
Add 50 µl of mixed donors and recipients to each well of the conjugation plate. Let dry 1 hour in a biological hood.
3-G
Cover with gas permeable film and let conjugate at 37 °C for 2 hours.
3-H
Add 400 µl of selection medium and ressuspend with agitation (900 rpm / 10 min.).
3-I
250 µl
Transfer 250 µl to a new deep-well filled with 1500 µl of selective medium and incubate overnight with agitation.
20 µl
3-J
Using the 20 µl of the remaining liquid, perform serial dilutions.
3-K
To quantify the efficiency of the mutagenesis, spot on selective media to obtain CFU counts of donor, recipients and transposon insertion mutants.
Day # 4
4-A
200 µl
Transfer 200 µl of culture to a new deep-well filled with selective medium.
Day # 5
5-A
100 µl
Transfer 100 µl of culture to a new deep-well filled with selective medium.
Day # 6
6-A
100 µl
Transfer 100 µl of culture to a new deep-well filled with selective medium.
Day # 7
7-A
150 µl
7-B
100 µl
Transfer 100 µl of culture to a new deep-well filled with selective medium. Use 150 µl to make a 25% glycerol stock of the culture in order to have backup for DNA extraction.
Day # 8
8-A
Centrifugation
4000 x g / 10 min.
8-B
Discard supernatant
Pellets ready for DNA extraction. Can be stored at -80 °C until extraction.

CIHAUB2E

WORKS FOR ME 1

HTTM : Extraction V.(cihaub2e)

Antoine Champie, Amélie De Grandmaison

Antoine Champie

COMMENTS 0

ABSTRACT

Part two of the HTTM protocol. A low-cost and high-throughput Tn-seq protocol.
This part cover the DNA extraction from cell pellets of transposon insertion mutants and subsequent silica columns regeneration.

PROTOCOL INFO

Antoine Champie, Amélie De Grandmaison . HTTM : Extraction. **protocols.io**
https://protocols.io/view/httm-extraction-cihaub2e

CREATED

Oct 27, 2022

LAST MODIFIED

Nov 05, 2022

PROTOCOL INTEGER ID

71938

MATERIALS TEXT

- **Homemade DNA lysis Buffer :**

A	B
Component	Amount for 1000 ml of solution
CTAB 2%	20 g
1.5 M Guanidine HCl	143.2 g
10 mM Tris HCl	1.57 g

Mix well and adjust volume to 1 l with water and adjust pH to 8.0.

- **Homemade wash solution :**

A	B
Component	Amount for 1000 ml of solution
Ethanol 100%	800 ml
Tris HCl 1 M pH 8.0	10 ml
NaCl 4 M	25 ml
EDTA 0.5 M	2 ml

Mix well and adjust volume to 1 l with water and adjust pH to 8.0.

- **Elution Buffer (Low TE Buffer)**: 10 mM Tris-HCl (pH 8.0) + 0.1 mM EDTA

Solutions for plate regeneration, from this protocol : (1)https://doi.org/10.1016/j.ab.2008.10.021.

- **NaOH 1N + Triton X-100 0.15% (v/v)**

A	B
Component	Amount for 1000 ml of solution
Water	960 ml
NaOH	40 g
Triton X-100	1.5 ml

Mix well and store in a base resistant container.

- **HCl 1.5N + Triton X-100 0.15% (v/v)**

A	B
Component	Amount for 1000 ml of solution
Water	873.5 ml
HCl Stock (37%)	125 ml

Triton X-100	1.5 ml

Mix well and store in an acid resistant container.

Silica columns array come from the following commercially available kit :
96-Well Plate Bacteria Genomic DNA Miniprep Kit from Biobasic. CAT#: SK1295

2h 5m

DNA extraction

1 Prepare the lysis solution by adding 165 µL of proteinase K to 66 mL of homemade lysis buffer and mix well.

2 Add 600 µL of lysis solution to each well of the deep-well plate and resuspend the pellet.

3 Cover with an adhesive aluminum cover and incubate at 55 °C for 01:00:00 . 1h

4 While still warm, add 260 µL of ethanol 100%, without overmixing.

Note

Overmixing will result in DNA agglomeration and difficulty with the extraction.

5 Transfer immediately to a deep-well plate fitted with an array of silica columns.

6 Centrifuge twice at 4000 x g, 00:10:00 . 10m

7 Discard flowthrough and add 500 µL of wash solution.

8 Centrifuge at 3000 x g, 00:10:00 . 10m

8.1 Repeat steps 7 and 8.

9 Discard flowthrough.

10 Centrifuge at 3000 x g, 00:10:00 to eliminate traces of wash solution. 10m

11 Discard flowthrough.

12 Add a collector plate between the silica column array and the deep-well plate.

13 Add 50 µL of low TE to the silica matrix in each well.

14 Cover with an adhesive aluminum foil and incubate at 55 °C for 00:15:00 . 15m

15 Centrifuge at 3000 x g, 00:10:00 . 10m

Silica array regeneration (Optional)

1h 5m

16 Put the contaminated silica array on an empty deep-well plate.

Add 150 µL of 1N NaOH + 0.15%(v/v) Triton X-100 to each well.

17 Incubate at Room temperature for 00:05:00 . 5m

18 Centrifuge 3000 x g, 00:05:00 . 5m

19 Add 200 µL of 1.5N HCl+ 0.15% (v/v) Triton X-100 to each well.

20 Incubate at Room temperature for 00:30:00 . 30m

21 Centrifuge 3000 x g, 00:05:00 . 5m

22 Add 150 µL of 1N NaOH + 0.15%(v/v) Triton X-100 to each well.

23 Incubate at Room temperature for 00:05:00 . 5m

24 Centrifuge 3000 x g, 00:05:00 . 5m

24.1 Collect the flowthrough in a beaker. Neutralize pH if needed and dispose of the flow through.

25 Add 200 µL of ddH_2O to each well.

26 Centrifuge 3000 x g, 00:05:00 . 5m

26.1 Repeat steps 25 and 26.

27 Silica columns array are ready to be reused.

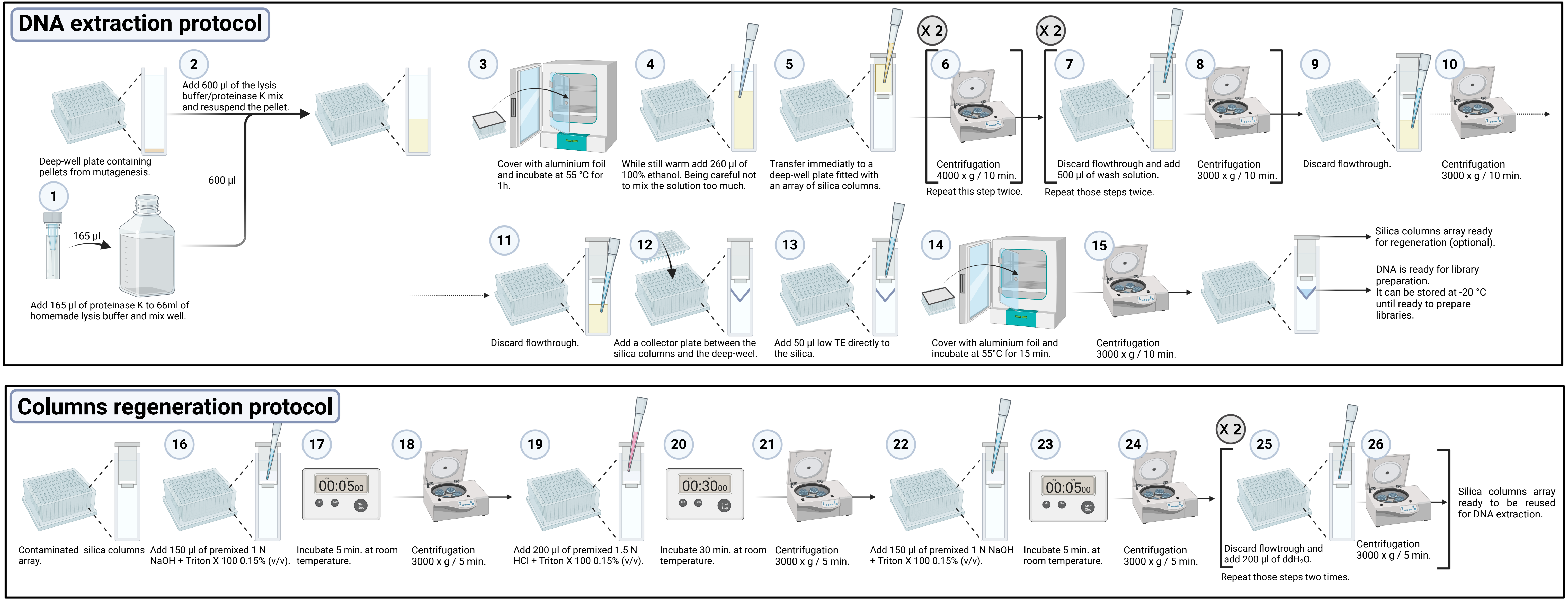
DNA extraction protocol
2
Add 600 µl of the lysis buffer/proteinase K mix and resuspend the pellet.
Deep-well plate containing pellets from mutagenesis.
600 µl
1
165 µl
Add 165 µl of proteinase K to 66ml of homemade lysis buffer and mix well.
3
Cover with aluminium foil and incubate at 55 °C for 1h.
4
While still warm add 260 µl of 100% ethanol. Being careful not to mix the solution too much.
5
Transfer immediately to a deep-well plate fitted with an array of silica columns.
X 2
6
Centrifugation 4000 x g / 10 min.
Repeat this step twice.
X 2
7
Discard flowthrough and add 500 µl of wash solution.
8
Centrifugation 3000 x g / 10 min.
Repeat those steps twice.
9
Discard flowthrough.
10
Centrifugation 3000 x g / 10 min.
11
Discard flowthrough.
12
Add a collector plate between the silica columns and the deep-weel.
13
Add 50 µl low TE directly to the silica.
14
Cover with aluminium foil and incubate at 55°C for 15 min.
15
Centrifugation 3000 x g / 10 min.
Silica columns array ready for regeneration (optional).
DNA is ready for library preparation.
It can be stored at -20 °C until ready to prepare libraries.
Columns regeneration protocol
Contaminated silica columns array.
16
Add 150 µl of premixed 1 N NaOH + Triton X-100 0.15% (v/v).
17
00:05 00
Incubate 5 min. at room temperature.
18
Centrifugation 3000 x g / 5 min.
19
Add 200 µl of premixed 1.5 N HCl + Triton X-100 0.15% (v/v).
20
00:30 00
Incubate 30 min. at room temperature.
21
Centrifugation 3000 x g / 5 min.
22
Add 150 µl of premixed 1 N NaOH + Triton-X 100 0.15% (v/v).
23
00:05 00
Incubate 5 min. at room temperature.
24
Centrifugation 3000 x g / 5 min.
X 2
25
Discard flowtrough and add 200 µl of ddH$_2$O.
26
Centrifugation 3000 x g / 5 min.
Repeat those steps two times.
Silica columns array ready to be reused for DNA extraction.

CIHFUB3N

WORKS FOR ME 1

HTTM : Libraries V.(cihfub3n)

Antoine Champie, Amélie De Grandmaison

Antoine Champie

COMMENTS 0

ABSTRACT

Part three of the HTTM protocol. A low-cost and high-throughput Tn-seq protocol.
This part cover the preparation of Illumina sequencing libraries form genomic DNA.

PROTOCOL INFO

Antoine Champie, Amélie De Grandmaison . HTTM : Libraries. **protocols.io**
https://protocols.io/view/httm-libraries-cihfub3n

CREATED

Oct 27, 2022

LAST MODIFIED

Nov 06, 2022

PROTOCOL INTEGER ID

71943

MATERIALS TEXT

Preparation of Nextera adapters :

Nextera (NxT) adapters are prepared by hybridization of the following primers :

A	B
Nxt-XTv2-B-N701-T	CAAGCAGAAGACGGCATACGAGATTCGCCTTAGTCTCGTGGGCTCGGAGATGTGTATAAGAGACAGT
Nxt-XTv2-B-3R-ac3-phos5'	/5Phos/CTGTCTCTTATACACATCTCCGAGCCCACGAGAC/3InvdT/

- **Preparation of the 5X annealing buffer (5X Tris NaCl buffer : 50 mM Tris, pH 7.5-8, 250 mM NaCl) :**
- 500 µl Tris-HCl 1M pH 7.5
- 500 µl NaCl 5M
- 9 ml H2O mol.-grade

- **Preparation of the adapters (40 µM 50 µL) :**
- Resuspend both primers in water to obtain 100 µM stocks
- Mix 20 µl of each (Nxt-XTv2-B-N701-T and Nxt-XTv2-B-3R-ac3-phos5')
- Add 10 µl of 5X annealing buffer
- Annealing reaction in a thermocycler (decrease temperature from 98 ℃ to 4 ℃ (-0.1 ℃/cycle(10s/cycle)))

Primers used for the first PCR :

A	B
Nxt_A	AATGATACGGCGACCACCGAGATCTACAC
Nxt_B	CAAGCAGAAGACGGCATACGAGAT

Primers template for barcoding PCR :

A	B
Nxt_i5_barcoding	AATGATACGGCGACCACCGAGATCTACAC [8 Nu Index] TCGTCGGCAGCGTCAGATGTGTA
Nxt_i7_barcoding	CAAGCAGAAGACGGCATACGAGAT [8 Nu Index] GTCTCGTGGGCTCGGAGATGTGTATAAG

Kit used for library preparation :
NEBNext Ultra II DNA Library Prep Kit for Illumina
NEB CAT#: E7645S

PCR mix used :
Supermix 2X
Homemade

SPRI beads used:
Ampure XP DNA beads
Beckman Coulter CAT#: A63882

BEFORE STARTING

- All steps and master mixes need to be kept on ice as much as possible. Thermocyclers need to be cooled at 4 ℃ before inserting sample plate.

1h 34m

Libraries

1 Transfer 2.5 µL of DNA from the DNA extraction plate to a new PCR plate.

2 Prepare a fragmentation master mix with :

A	B
NEB Ultra II FS buffer	77 µl
NEB Ultra II FS enzyme	22 µl
Molecular grade water	11 µl

3 Add 1 µL of the fragmentation master mix to each well.

45m

4 Incubate in a thermocycler with the following protocol :

- 00:15:00 at 37 °C
- 00:30:00 at 65 °C

5 Add 1 µL of 4µM Nextera (NxT) adaptors to each well.

6 Prepare a ligation master mix with :

A	B
NEB Ultra II ligation master mix	377.4 µl
NEB Ultra II ligation enhancer	12.1 µl

7 Add 3.5 µL of ligation master mix to each well.

40m

8 Incubate in a thermocycler with the following protocol :

- 00:30:00 at 20 °C
- 00:10:00 at 65 °C

9 Prepare a PCR master mix with :

A	B
NxT_A primer 20 µM	880 µl
Nxt_B primer 20 µM	880 µl
Molecular grade water	8360 µl
PCR Supermix 2X	11000 µl

10 Add 192 µL of PCR master mix to each well.

11 Split the PCR reaction into 4 different plates (50µl per plate).

12 Incubate each plate in a thermocycler with the following cycles : 3m 15s

- 00:00:30 at 98 °C
- 00:00:15 at 98 °C
- 00:00:30 at 72 °C
- Repeat from step 2 for 20~25 cycles*
- 00:02:00 72 °C

13 Pool the 4 PCR replicates together in a PCR plate.

14 Transfer 2 µL of DNA from the pool plate to a new PCR plate.

15 Add 2 µL of each barcoding primer to the DNA :

- Nxt_i5_barcoding
- Nxt_i7_barcoding

16 Prepare a PCR master mix with :

A	B
Molecular grade water	2090 µl
PCR supermix 2X	2750 µl

17 Add 44 µL of the PCR master mix to each well of the plate.

18 Incubate in a thermocycler using the following protocol : 3m 45s

- 00:00:30 at 98 °C
- 00:00:15 at 98 °C
- 00:01:00 at 72 °C (no anneal step)
- Repeat from step 2 for 5 cycles
- 00:02:00 at 72 °C

19 Pool together 2 µL of each sample.

20 Purify with Ampure XP SPRI beads using a 0.8 ratio. Resuspend with 50 µL of molecular grade water.

21 Proceed with QC and sequencing.

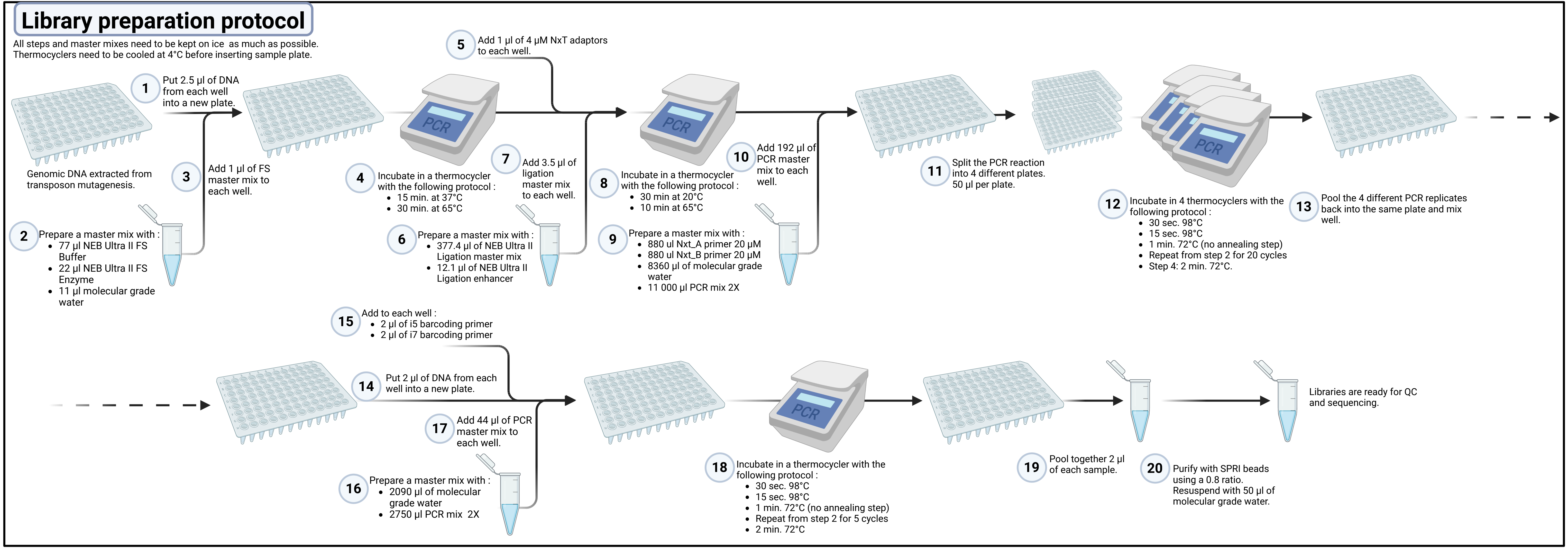
Library preparation protocol
All steps and master mixes need to be kept on ice as much as possible.
Thermocyclers need to be cooled at 4°C before inserting sample plate.
Genomic DNA extracted from transposon mutagenesis.
1 Put 2.5 µl of DNA from each well into a new plate.
2 Prepare a master mix with : 77 µl NEB Ultra II FS Buffer; 22 µl NEB Ultra II FS Enzyme; 11 µl molecular grade water
3 Add 1 µl of FS master mix to each well.
PCR
4 Incubate in a thermocycler with the following protocol : 15 min. at 37°C; 30 min. at 65°C
5 Add 1 µl of 4 µM NxT adaptors to each well.
6 Prepare a master mix with : 377.4 µl of NEB Ultra II Ligation master mix; 12.1 µl of NEB Ultra II Ligation enhancer
7 Add 3.5 µl of ligation master mix to each well.
8 Incubate in a thermocycler with the following protocol : 30 min at 20°C; 10 min at 65°C
9 Prepare a master mix with : 880 ul Nxt_A primer 20 µM; 880 ul Nxt_B primer 20 µM; 8360 µl of molecular grade water; 11 000 µl PCR mix 2X
10 Add 192 µl of PCR master mix to each well.
11 Split the PCR reaction into 4 different plates. 50 µl per plate.
12 Incubate in 4 thermocyclers with the following protocol : 30 sec. 98°C; 15 sec. 98°C; 1 min. 72°C (no annealing step); Repeat from step 2 for 20 cycles; Step 4: 2 min. 72°C.
13 Pool the 4 different PCR replicates back into the same plate and mix well.
14 Put 2 µl of DNA from each well into a new plate.
15 Add to each well : 2 µl of i5 barcoding primer; 2 µl of i7 barcoding primer
16 Prepare a master mix with : 2090 µl of molecular grade water; 2750 µl PCR mix 2X
17 Add 44 µl of PCR master mix to each well.
18 Incubate in a thermocycler with the following protocol : 30 sec. 98°C; 15 sec. 98°C; 1 min. 72°C (no annealing step); Repeat from step 2 for 5 cycles; 2 min. 72°C
19 Pool together 2 µl of each sample.
20 Purify with SPRI beads using a 0.8 ratio. Resuspend with 50 µl of molecular grade water.
Libraries are ready for QC and sequencing.

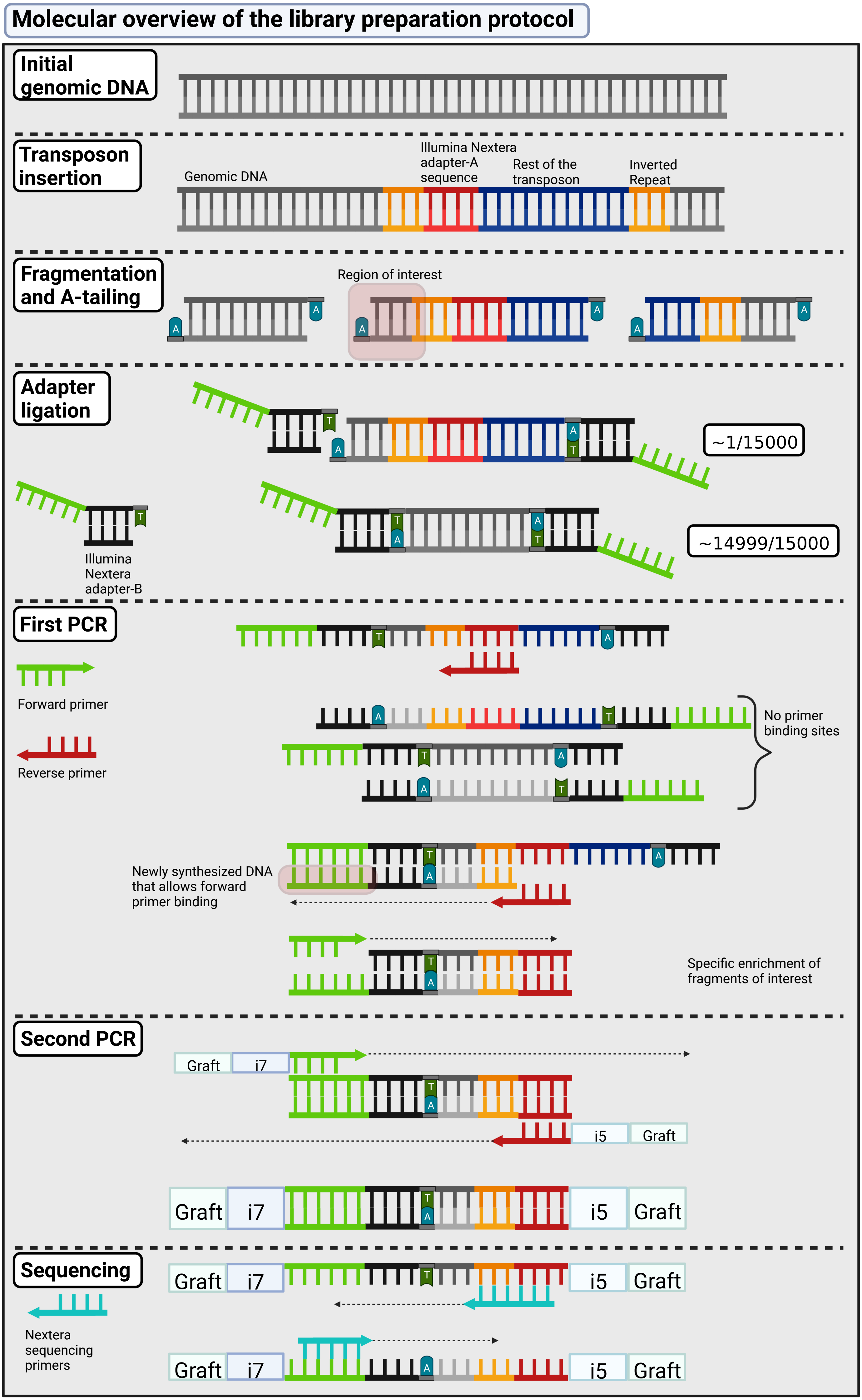
Molecular overview of the library preparation protocol
Initial genomic DNA
Transposon insertion
Genomic DNA
Illumina Nextera adapter-A sequence
Rest of the transposon
Inverted Repeat
Fragmentation and A-tailing
Region of interest
Adapter ligation
~1/15000
~14999/15000
Illumina Nextera adapter-B
First PCR
Forward primer
Reverse primer
No primer binding sites
Newly synthesized DNA that allows forward primer binding
Specific enrichment of fragments of interest
Second PCR
Graft
i7
i5
Graft
Sequencing
Nextera sequencing primers
