# Supplementary material for: Enabling low-cost and robust essentiality studies with high-throughput transposon mutagenesis (HTTM)
Source: PLoS One. 2023 Apr 11;18(4):e0283990. doi: 10.1371/journal.pone.0283990 (PMC10089323; doi:10.1371/journal.pone.0283990)
Supplement: S6 File — (PDF) [file pone.0283990.s006.pdf]

Enabling low-cost and robust essentiality studies with high-throughput transposon mutagenesis (HTTM)

Supplementary figures

List of supplementary figures:

- **Figure S1: Concentration of DNA recovered after successive extractions and regenerations of silica columns**
- **Figure S2: Test of silica columns contamination between successive genomic DNA extractions**
- **Figure S3: DNA extraction tests using different homemade solutions**
- **Figure S4: Specificity test of the first PCR step of library preparation using either wild-type DNA or transposon-mutated DNA**
- **Figure S5: Influence of the heat-inactivated ligation mix on the first PCR amplification**
- **Figure S6: Example of the bimodal insertion index distribution in a replicate**
- **Figure S7: BW25113 genomic locus showing representative insertion densities across different passages for essential (*ligA*, *zipA*, *ptsH*, and *ptsI*) and non-essential genes**
- **Figure S8: Average number of insertion sites per sample as a function of the number of pooled PCR replicate**
- **Figure S9: Example of genes presenting variations in the essentiality calls across replicates caused by a small number of insertions**

  Figure S10: Example of genes displaying a low insertion density at passage 5

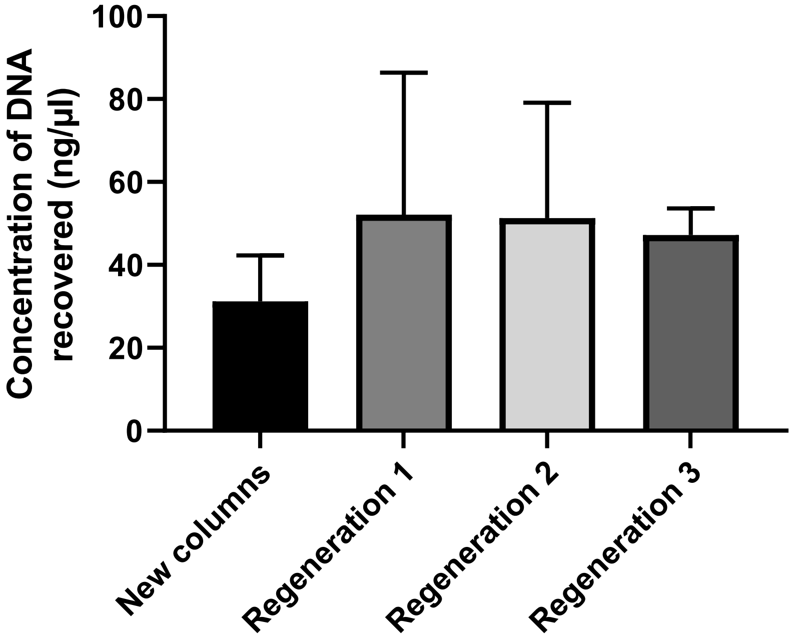

Figure S1: Concentration of DNA recovered after successive extractions and regenerations of silica columns. The box and whiskers indicate the mean and standard deviation calculated from technical triplicates. DNA concentration was measured using the Quant-iT PicoGreen dsDNA Assay Kit (ThermoFisher).

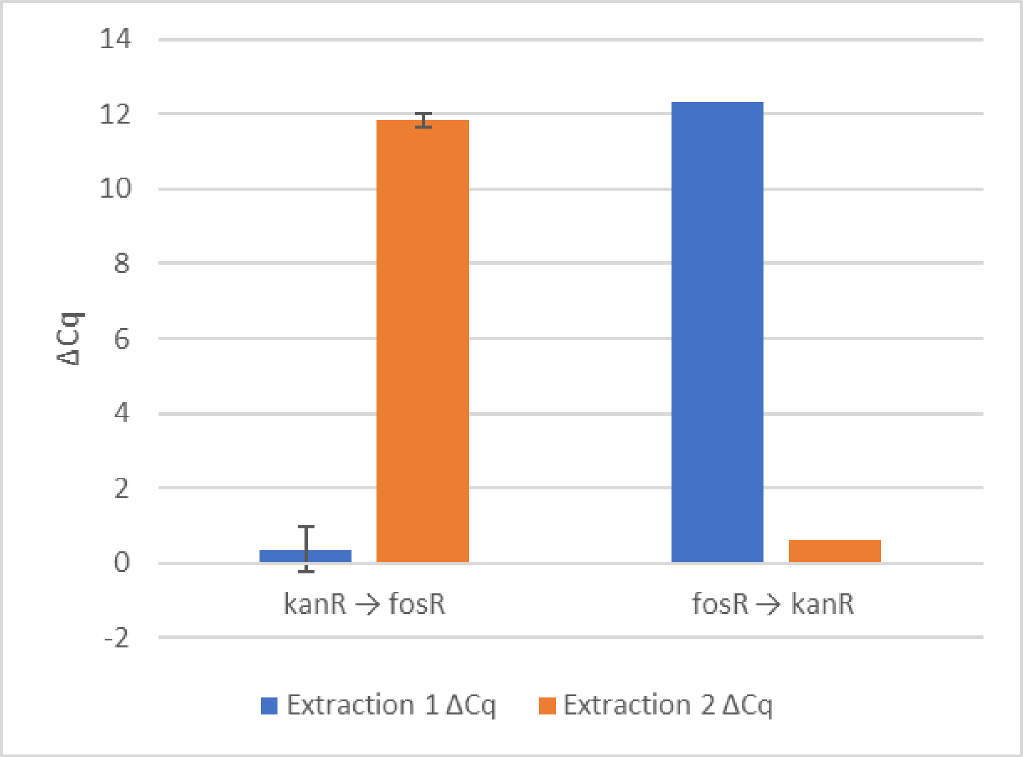

Figure S2: Test of silica columns contamination between successive genomic DNA extractions. DNA from two distinct strains of *E. coli*, one bearing the kanamycin resistance (kanR) marker and the other bearing the fosfomycin resistance (*fosR*) marker have been extracted using two different silica columns. In the first column the *kanR*-tagged genomic DNA was extracted, the column was regenerated and the *fosR*-tagged genomic DNA was extracted. In the second column, the extraction order was reversed. DNA from all four extractions was then used as a quantitative PCR template with primers targeting a common region of the genome as a control and primers specific to the *kanR* marker. ΔC_q represents the difference between the C_q of the *kanR* amplification minus the C_q of the control, which should be near zero in case of the presence of the *kanR* gene and around 12 (our detection limit) in its absence. IQ SYBR Green Super mix was used for all amplifications. Amplifications were monitored using a CFX Connect Real-Time PCR Detection System (Bio-Rad).

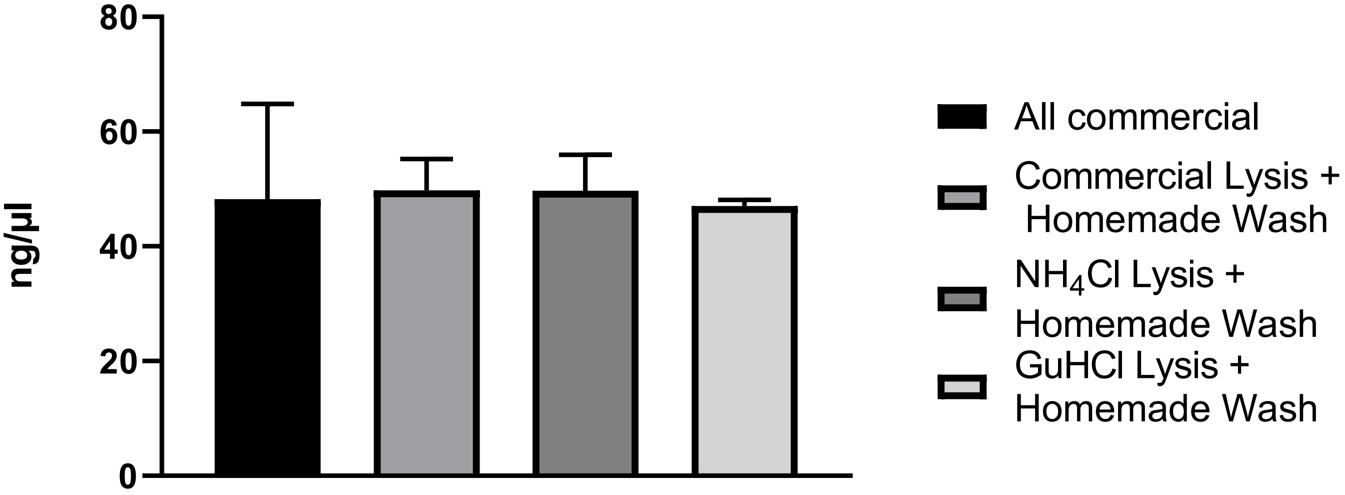

Figure S3: DNA extraction tests using different commercial or homemade solutions. Genomic DNA purification was performed using the 96-Well Plate Bacteria Genomic DNA Miniprep Kit from Biobasic (Cat. #: SK1295). The box and whiskers indicate the mean and standard deviation calculated from technical triplicates. GuHCl, guanidine HCl. Recipe for guanidine HCl (GuHCl) lysis buffer (CTAB 2%, 1.5M GuHCl, 10mM Tris HCl, pH 8.0). Recipe for NH_4Cl lysis buffer (CTAB 2%, 1.5M NH_4Cl, 10mM Tris HCl, pH 8.0). Recipe for homemade wash solution (Ethanol [80% final volume], 10 mM Tris HCl, NaCl 100 mM, EDTA 1 mM (ml), pH 8.0). DNA concentration was measured using the Quant-iT PicoGreen dsDNA Assay Kit (ThermoFisher).

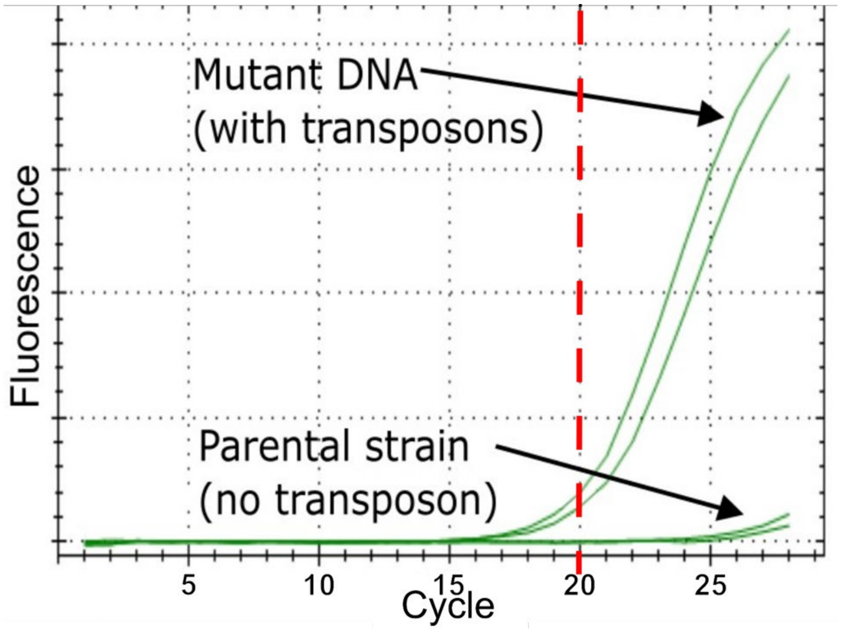

Figure S4: Specificity test of the first PCR step of library preparation using either wild-type DNA or transposon-mutated DNA. Cycle 20 at which the reaction is stopped in the HTTM protocol is indicated by a red dashed line. Amplification was performed using the Supermix 2X (Homemade) and monitored using a CFX Connect Real-Time PCR Detection System (Bio-Rad).

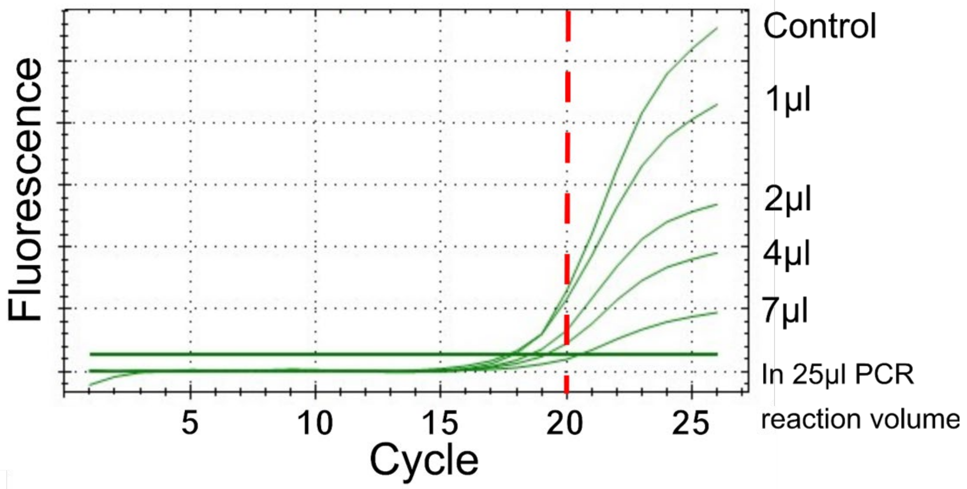

Figure S5: Influence of the heat-inactivated ligation mix on the first PCR amplification. Quantitative PCR amplification curves were obtained using a variable volume of heat-inactivated ligation mix for the preparation of Illumina sequencing libraries. While the final DNA concentration is affected, the volume added does not affect the C_q of the reaction. In the HTTM protocol, amplification is stopped at cycle 20 (red dashed line). Control; no heat-inactivated ligation mix added to the PCR reaction. PCR reactions were all prepared with 5 ng of ligated and purified DNA, a variable volume of heat-inactivated ligation mix, 12.5 µl of 2X Supermix (Homemade), and completed up to 25 µl with molecular grade water. While the addition of 1 µl of heat-inactivated ligation mix slightly lowers the final concentration of DNA obtained, which is of low impact since we stop the PCR at cycle 20 (red dashed line), it does not affect the C_q of the reaction and thus has been determined as an acceptable amount of contamination in our PCR reactions. These tests have been performed with a final reaction volume of 25 µl and the ratio of heat-inactivated ligation mix is kept when using 50 µl PCR reactions (i.e., 2 µl in 50 µl reactions).

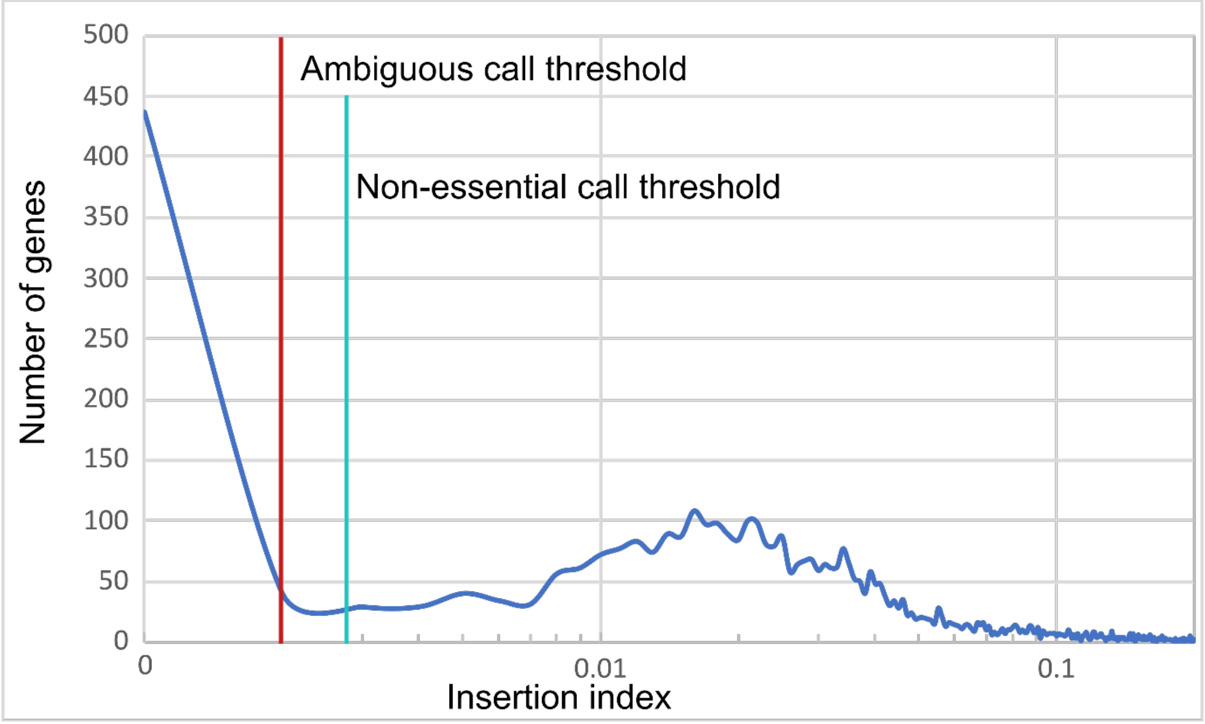

Figure S6: Example of the bimodal insertion index distribution in a replicate. Thresholds are automatically determined by the Bio-Tradis toolkit. Genes with an insertion index under the ambiguous call threshold are flagged as essentials.

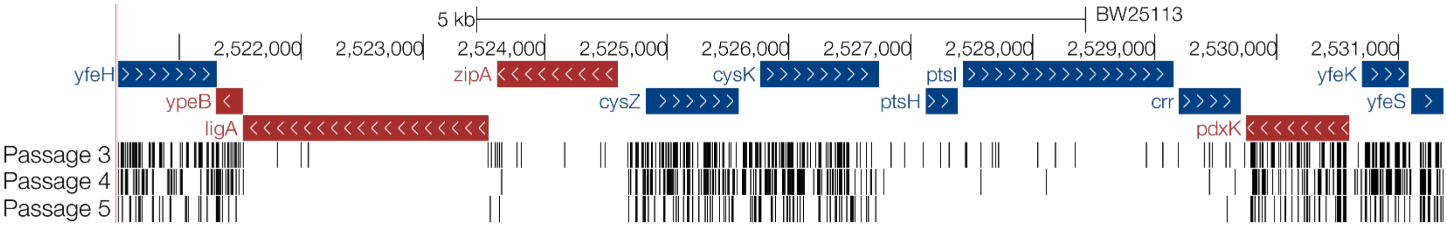

Figure S7: BW25113 genomic locus showing representative insertion densities across different passages for essential (*ligA*, *zipA*, *ptsH*, and *ptsI*) and non-essential genes. Genes are color-coded according to their orientation.

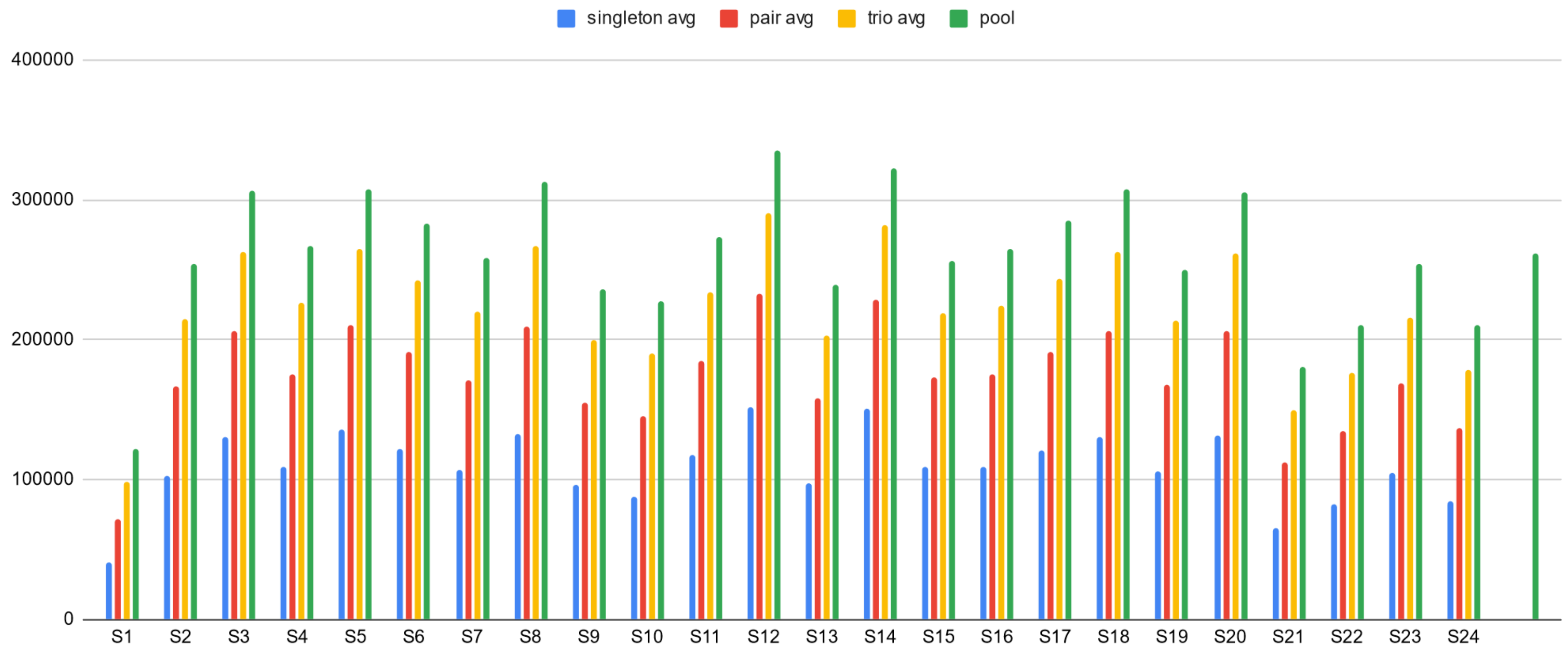

Figure S8: Average number of insertion sites per sample as a function of the number of pooled PCR replicates.

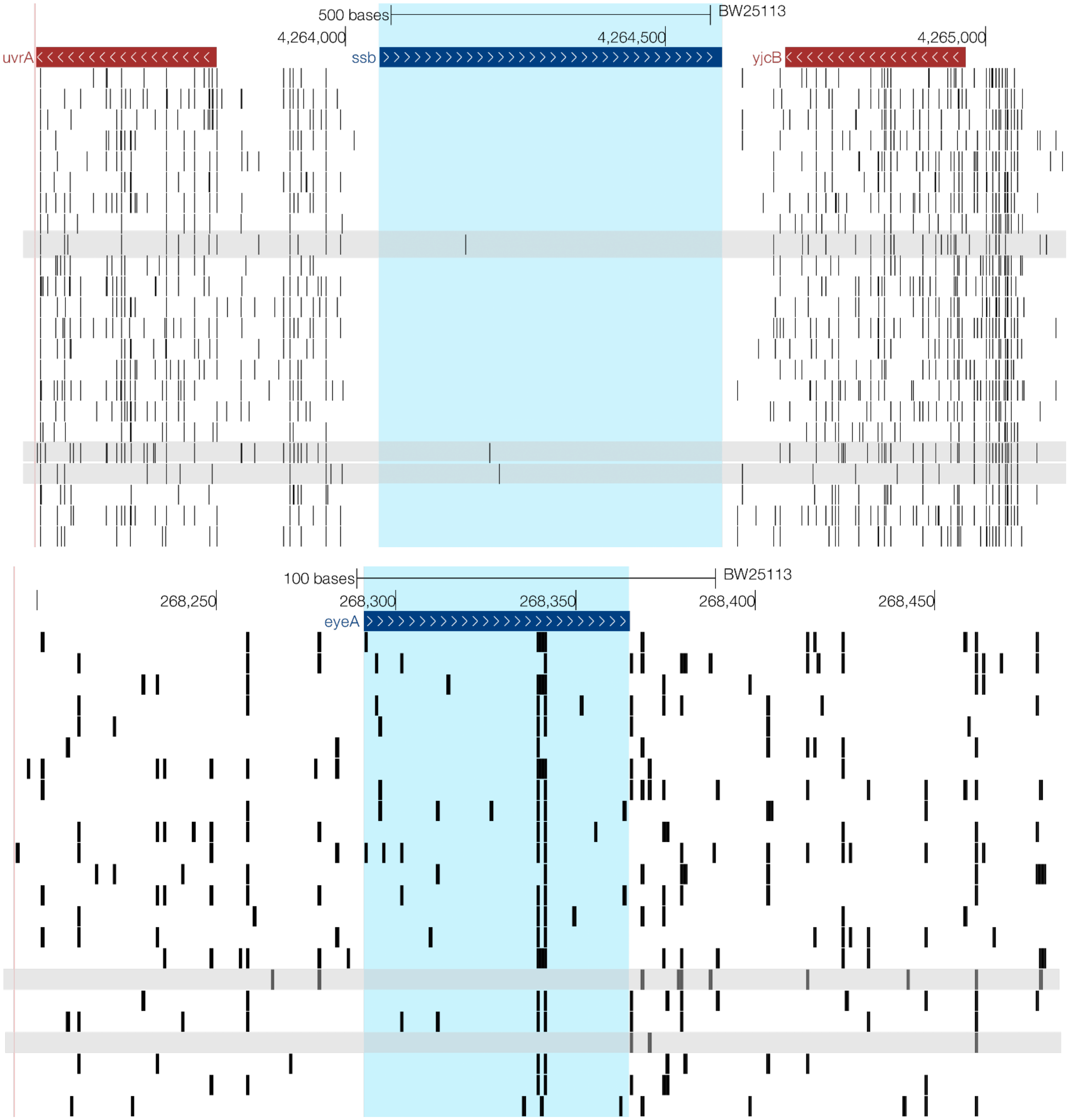

Figure S9: Example of genes presenting variations in the essentiality calls across replicates caused by a small number of insertions. Each row is a different replicate at passage 5. Gray shadings indicate replicates whose essentiality status called by the Biotradis Toolkit deviates from the consensus. Genes are color-coded according to their orientation.

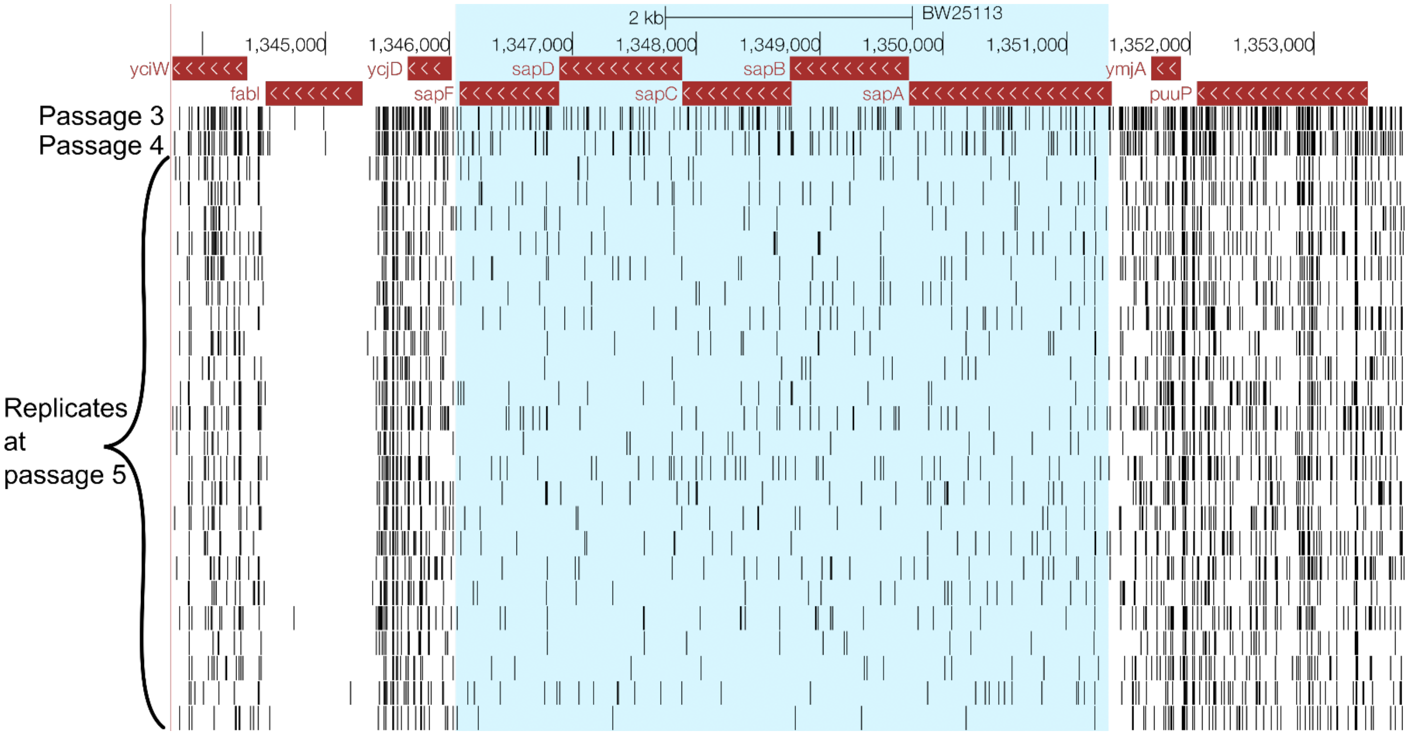

Figure S10: Example of genes displaying a low insertion density at passage 5. Insertion mutants showing interruptions within the *sapABCDF* operon (blue shading) are slowly lost during passages as a result of a lower fitness and competition with the population, resulting in low insertion density at passage 5 and variable essentiality calls.
